# Association of Roadway Proximity with Indoor Air Pollution in a Peri-Urban Community in Lima, Peru

**DOI:** 10.3390/ijerph121013466

**Published:** 2015-10-26

**Authors:** Lindsay J. Underhill, Sonali Bose, D’Ann L. Williams, Karina M. Romero, Gary Malpartida, Patrick N. Breysse, Elizabeth M. Klasen, Juan M. Combe, William Checkley, Nadia N. Hansel

**Affiliations:** 1 Department of Environmental Health, Boston University School of Public Health, Boston, MA 02118, USA; E-Mail: lju@bu.edu; 2 Division of Pulmonary and Critical Care, School of Medicine, John Hopkins University, Baltimore, MD 21224, USA; E-Mails: sbose7@jhmi.edu (S.B.); emklasen@gmail.com (E.M.K.); wcheckl1@jhmi.edu (W.C.); 3 Department of Environmental Health Sciences, Bloomberg School of Public Health, Johns Hopkins University, Baltimore, MD 21205, USA; E-Mails: dwilli20@jhu.edu (D.L.W.); pbreysse@jhsph.edu (P.N.B.); 4 Center for Asthma Research, A.B. PRISMA, Lima 32, Peru; E-Mails: kromero@prisma.org.pe (K.M.R.); jmanuelcombe@gmail.com (J.M.C.); 5 Laboratorio de Investigacion y Desarrollo, Universidad Peruana Cayetano Heredia, Lima 31, Peru; E-Mail: gary21mg@gmail.com; 6 Program in Global Disease Epidemiology and Control, Bloomberg School of Public Health, Johns Hopkins University, Baltimore, MD 21205, USA

**Keywords:** air pollution, indoor environment, particulate matter, black carbon, nitrogen dioxide, allergens, asthma, traffic, childhood, low-income and vulnerable populations

## Abstract

The influence of traffic-related air pollution on indoor residential exposure is not well characterized in homes with high natural ventilation in low-income countries. Additionally, domestic allergen exposure is unknown in such populations. We conducted a pilot study of 25 homes in peri-urban Lima, Peru to estimate the effects of roadway proximity and season on residential concentrations. Indoor and outdoor concentrations of particulate matter (PM_2.5_), nitrogen dioxide (NO_2_), and black carbon (BC) were measured during two seasons, and allergens were measured in bedroom dust. Allergen levels were highest for dust mite and mouse allergens, with concentrations above clinically relevant thresholds in over a quarter and half of all homes, respectively. Mean indoor and outdoor pollutant concentrations were similar (PM_2.5_: 20.0 *vs.* 16.9 μg/m^3^, BC: 7.6 *vs.* 8.1 μg/m^3^, NO_2_: 7.3 *vs.* 7.5 ppb), and tended to be higher in the summer compared to the winter. Road proximity was significantly correlated with overall concentrations of outdoor PM_2.5_ (r_s_ = −0.42, *p* = 0.01) and NO_2_ (r_s_ = −0.36, *p* = 0.03), and outdoor BC concentrations in the winter (r_s_ = −0.51, *p* = 0.03). Our results suggest that outdoor-sourced pollutants significantly influence indoor air quality in peri-urban Peruvian communities, and homes closer to roadways are particularly vulnerable.

## 1. Introduction

Air pollution, including fine particulate matter (PM_2.5_), black carbon (BC), and nitrogen dioxide (NO_2_) [1], is widely recognized for its impact on cardiopulmonary disease, and has been of increasing concern in the developing world [2]. Traffic-related sources of these pollutants may be particularly significant contributors to poor air quality due to rapidly expanding metropolitan areas and concomitant surges in traffic and pollution-generating fuel usage [3,4]. While much of the existing literature on traffic-related pollution has focused on urban centers of high-income countries, less is known regarding the extent to which these pollutants contribute to the indoor exposures of residents living in lower-income regions. Moreover, unique exposure patterns in developing areas related to housing construction characteristics (e.g., building tightness and ventilation patterns) and residential activities (e.g., cooking, smoking) may be distinct from industrialized nations where previous work has focused [5]. Ultimately, the burden of such traffic-related exposures may be greater in underdeveloped areas due to a relative paucity in pollution regulation as well as limitations in health care availability [2]. However, despite these environmental concerns, only a few studies have characterized the impact of traffic-related pollution in developing regions [4,6,7,8,9], and even fewer have explored its influence on the indoor environment of residential homes [10].

To address these gaps, we first conducted a pilot study to estimate the effect of roadway proximity on indoor pollutant concentrations in peri-urban communities with a high population density and associated traffic in Lima, Peru. This pilot study is part of a larger investigation into the impact of environmental and genetic factors on childhood asthma in this region. Furthermore, given the critical role of allergen exposure in the development and morbidity of certain phenotypes of asthma, as well as the relative lack of published data on allergen exposure in asthmatic children in Peru, we also explored in-home concentrations of common allergens. An improved understanding of the contributors to indoor environmental pollution in these homes is essential to developing future strategies aimed at reducing their burden on the health of these populations.

## 2. Experimental Section

### 2.1. Study Setting

Pampas de San Juan de Miraflores and Villa El Salvador, two densely settled, neighboring peri-urban communities located 20 km south of the center of Lima, Peru, were chosen as study sites. Roads in these regions include both paved and unpaved surfaces with varying levels of traffic, primarily diesel-fueled commuter buses and motorbike taxis, and secondarily, gas-fueled taxis and personal motor vehicles.

### 2.2. Study Homes

Twenty-five homes were recruited across both Pampas de San Juan de Miraflores and Villa El Salvador at varying levels of elevation and distance from main roads. The selected network of main roads was based on local knowledge and a qualitative inspection of traffic density. Inclusion criteria for the homes were as follows: safe access to the roof and availability of a secure area for the operation of sampling machines; available outlets and presence of reliable electric current to the home; and, no plans to perform construction in or around the home for the entire seven-day sampling period. Residents of these homes were provided with informed consent and the study was approved by the institutional review boards of Johns Hopkins Bloomberg School of Public Health in Baltimore, USA and A.B. PRISMA in Lima, Peru.

### 2.3. Home Inspection and Questionnaires

Structural housing characteristics (e.g., size, building materials, number of rooms and windows, *etc.*) and in-home exposures (e.g., fuel sources for cooking and presence of pets) were assessed using an interviewer-administered questionnaire and inspection form. Additional qualitative information, such as the cleanliness of the dwelling and evidence of smoking or trash burning was based on subjective evaluation by the field interviewer. Participants were asked to complete a standardized daily activity diary for each day of sampling that recorded time spent indoors and home activities such as cleaning, cooking, and smoking.

### 2.4. Pollutant Monitoring

Indoor and outdoor concentrations of PM_2.5_, NO_2_, BC, temperature, and humidity were measured for two, non-sequential, seven-day periods in all participant homes during the summer and winter seasons. Round 1 sampling occurred from January to early June (summer), and Round 2 sampling occurred from late June through September (winter). During the first monitoring period, simultaneous sampling was performed in three separate areas of the home: (1) the roof in protective weatherproof housing; (2) the participant bedroom; and (3) the living room. For the second round of monitoring, the living room was not sampled due to the observed lack of variation between several living room and bedroom samples, the inconsistent presence and type of living room, and the predominant amount of indoor time spent within the bedroom based on informal participant reports (data not shown); therefore only results for pollutant concentrations in the bedroom are reported.

Particulate matter was collected on Teflon, PTFE filters (Pall Corporation, Port Washington, NY, USA) using the pDR-1200 (Thermo Scientific, Franklin, MA, USA) equipped with a size-selective cyclone inlet and vacuum pump BGI 400S (BGI Inc., Waltham, MA, USA) operating at the required flow rate of 4 LPM to capture the PM_2.5_ size fraction. Filters were pre- and post-weighed with a Mettler-Toledo MT5 microbalance under temperature and humidity controlled conditions at the Johns Hopkins Bloomberg School of Public Health using accepted EPA Federal Reference Methods [11]. All gravimetric PM_2.5_ results were temperature and humidity corrected using data collected with a HOBO data logger (Onset Computer Corporation, Bourne, MA, USA) [12].

BC was assessed using Teflon PM_2.5_ filters collected using the pDR 1200 and analyzed at the Johns Hopkins Bloomberg School of Public Health using the Magee OT-21 SootScan™ Model Transmissometer (Magee Scientific Corporation, Berkeley, CA, USA).

NO_2_ sampling was conducted using passive sampling monitors (Ogawa and Company Inc., Pompano Beach, FL, USA) loaded with triethanolamine (TEA)-coated filters, as previously described [13]. Duplicate sampling was performed in a subset of homes. Analysis was conducted at the Universidad Peruana Cayetano Heredia in Lima, Peru using established Ogawa protocols [14].

### 2.5. Allergen Dust Collection

Household dust samples were collected from the bed and bedroom floor and assayed for dog (Can f 1), cat (Fel d 1), mouse (Mus m 1), dust mite (Der p 1), and cockroach (Bla g 1) allergens in the laboratories of the Universidad Peruana Cayetano Heredia in Lima, Peru. Dust samples were collected using DUSTREAM^®^ nylon mesh filters (INDOOR Biotechnologies, Charlottesville, VA, USA) that were inserted into a portable vacuum (AB Electrolux, Stockholm, Sweden) using a DUSTREAM^®^ adaptor. The bed sample was collected by vacuuming the mattress and bedding for approximately 3 min and the floor sample was collected by vacuuming a square meter area under and around the bed, for approximately 2 min. After sampling, the adaptor and enclosed filter were sealed in a plastic Ziploc bag and stored at −30 °C until analysis. Each dust sample was sieved (sieve size, 300 µm) and an aqueous extract of each dust sample was prepared using phosphate buffered saline and stored at 30 °C until assayed using antibody-based ELISA methods previously described [15]. The analytical detection limits for Can f 1, Fel d 1, Mus m 1, Der p 1, and Bla g 1 were 4.12 ng/g, 1.66 ng/g, 0.66 ng/g, 9.88 ng/g, and 0.04 U/g, respectively.

### 2.6. Biostatistical Methods

Descriptive analyses were performed with means, medians, and proportions, as appropriate. Wilcoxon signed-rank tests (paired difference tests) were used to compare pollutant concentrations across seasons and sampling locations, and Wilcoxon rank sum tests were conducted to assess differences between pet allergen concentrations by the reported presence of residential dogs and cats. We calculated spearman correlations and used the Bland-Altman [16] plot to assess the agreement between outdoor and indoor pollutant concentrations. Spearman correlation tests were performed to assess the relationship between residential pollutants and distance to the nearest main road, which was calculated using ArcGIS 10 software.

The percent of households with detectable levels of dog, cat, mouse, and cockroach allergen were reported using multiple threshold categories previously defined by Curtin-Brosnan *et al.* [17]. The low threshold limits were defined by allergen-specific limits of detection (LODs) (detailed in Table 1 notes) while the medium and high threshold values for dog, cat, mouse, and cockroach allergens were based on their associations with increased risk of allergic sensitization and asthma symptoms, respectively, from prior literature selected by Curtin-Brosnan *et al.* [17]. Using this methodology, we determined threshold values for mite allergen based on the LOD and clinically relevant values from prior literature [18].

**Table 1:** Participant home characteristics.

Housing Characteristics	Mean	Min	Max
Household distance to nearest main road (m)	148.6	34.8	358.0
% Households less than 100 m from main road	36%		
Total rooms per home	7.2	3	14
Total bedrooms per home	3.7	1	8
Total windows per home	5.2	1	15
No. windows facing road	2.4	0	5
No. doorways to outside	1.9	0	5
% Bedrooms without window	32%		
% Homes with cleanliness ratings: *			
Below average	10%		
Average	57%		
Above average	33%		
Principal material of roof			
Iron or Tin	60%		
Cement	36%		
Brick	4%		
Principal material of residential walls			
Cement	52%		
Iron	24%		
Wood	16%		
Brick	8%		
% Homes that cook indoors by fuel type			
Gas	70.8%		
Charcoal	8.3%		
Electricity	8.3%		
Firewood	4.2%		
Unreported	8.4%		
% Indoor kitchens with ventilation	76%		
% Homes with pets/animals indoors			
Dog	42%		
Cat	16%		
Bird	16%		
Chicken	8%		

Notes: * Cleanliness rating scored by field technician.

All biostatistical analyses were conducted using R (R Foundation for Statistical Computing, www.r-project.org) and SAS 9.3 (SAS Institute Inc., Cary, NC, USA) statistical software.

## 3. Results

### 3.1. Household Characteristics

Participant homes had an average of seven rooms, with a range from 3 to 14 (Table 1). Over half of homes were rated as having an average level of cleanliness, with only 10% below the average. Natural gas was found to be the primary cooking fuel (70.8%), with less than 10% of homes reporting the use of solid fuels or electricity as supplemental energy sources. No evidence of indoor heating or cigarette smoking was reported by participants or observed by field interviewers. Household proximity to the main road ranged from 35 to 358 m. There were no statistically significant differences in housing characteristics between homes near (<100 m) or far from the road (>100 m) (data not shown).

### 3.2. Outdoor Environmental Exposures

The summer season was characterized by higher ambient temperature compared to the winter season (mean: 23 °C *vs.* 17 °C), as well as lower relative humidity (mean: 71% RH *vs.* 83% RH). Overall, mean outdoor concentrations of PM_2.5,_ BC and NO_2_ were 16.9 μg/m^3^ (range: 8.5–40.5 μg/m^3^), 8.1 μg/m^3^ (range: 3.6–19.3 μg/m^3^) and 7.5 ppb (4.2–14.0 ppb), respectively (Table 2). Mean outdoor concentrations of PM_2.5_ and BC were significantly higher in the summer compared to the winter (20.2 μg/m^3^ *vs.* 14.3 μg/m^3^, *p* < 0.001, and 9.3 μg/m^3^ *vs.* 7.0 μg/m^3^, *p* < 0.01, respectively) (Table 2). Average outdoor concentrations of NO_2_ also tended to be higher in the summer compared to winter (8.1 ppb *vs.* 7.1 ppb, *p* = 0.08); however this seasonal difference did not reach statistical significance (Table 2).

**Table 2:** Pollutant concentrations by season and sampling location.

Pollutant	Indoor	Mean (Range)	Outdoor	Mean (Range)	I *vs.* O *
	*n*		*n*		(*p*-Value)
PM_2.5_ (μg/m^3^)	39	20.0 (5.7–55.4)	42	16.9 (8.5–40.5)	0.14
Summer	20	20.8 (5.7–32.8)	19	20.2 (11.2–40.5)	0.47
Winter	19	19.2 (9.1–55.4)	23	14.3 (8.5–21.5)	0.23
S *vs.* W *	(*p*-value)	0.21		<0.001	
BC (μg/m^3^)	39	7.6 (3.5–21.1)	35	8.1 (3.6–19.3)	0.15
Summer	21	9.0 (4.9–21.08)	16	9.3 (4.1–19.3)	0.30
Winter	18	5.9 (3.5–10.3)	19	7.0 (3.6–15.1)	0.46
S *vs.* W *	(*p*-value)	0.01		<0.01	
NO_2_ (ppb)	34	7.3 (2.4–14.61)	36	7.5 (4.2–14.0)	0.69
Summer	16	8.5 (5.4–14.6)	15	8.1 (4.7–14.0)	0.45
Winter	18	6.2 (2.4–10.7)	21	7.1 (4.2–11.6)	0.26
S *vs.* W *	(*p*-value)	<0.001		0.08	

Notes: * Wilcoxon signed-rank test used to test differences between indoor and outdoor (I *vs.* O) and summer and winter (S *vs.* W) values.

There was a significant correlation between road proximity and outdoor PM_2.5_ (r_s_= −0.42, *p* = 0.01), as well as outdoor NO_2_ (r_s_ = −0.36, *p* = 0.03) (Table 3). The relationship between road proximity and PM_2.5_ seemed to be stronger in the summer compared to the winter (r_s_ = −0.71, *p* = 0.001 *vs.* r_s_ = −0.44, *p* = 0.04); in contrast, the correlation between NO_2_ and distance from the road was only slightly higher in the summer season (r_s_ = −0.49, *p* = 0.07 *vs.* r_s_ = −0.34, *p* = 0.14). Outdoor BC concentrations tended to be lower with further distance from the road, especially in the winter (r_s_ = −0.51, *p* = 0.03) compared to summer (r_s_ = 0.10, *p* = 0.70).

**Table 3:** Correlation between outdoor residential concentrations of PM_2.5_, BC, and NO_2_ and distance from the nearest major roadway.

Pollutant	*n*	r_s_	*p*-Value
**PM_2.5_**	42	**−0.42**	**0.01**
Summer	19	**−0.71**	**0.001**
Winter	23	**−0.44**	**0.04**
**BC**	35	−0.24	0.16
Summer	16	0.10	0.70
Winter	19	**−0.51**	**0.03**
**NO_2_**	36	**−0.36**	**0.03**
Summer	15	−0.49	0.07
Winter	21	−0.34	0.14

Notes: Results significant at the 0.05 level are bolded.

### 3.3. Indoor Environmental Exposures

Mean indoor concentrations of PM_2.5_, BC, and NO_2_ were 20.0 μg/m^3^ (range: 5.7 to 55.4 μg/m^3^), 7.6 μg/m^3^ (range: 3.5 to 21.1 μg/m^3^), and 7.3 ppb (range: 2.4–14.6), respectively (Table 2). Mean indoor concentrations of BC and NO_2_ were significantly higher in the summer compared to the winter (BC: 9.0 μg/m^3^ *vs.* 5.9 μg/m^3^, *p* < 0.01; NO_2_: 8.5 ppb *vs.* 6.2 ppb, *p* < 0.001) (Table 2). Meanwhile, average indoor concentrations of PM_2.5_ did not significantly vary by season (20.8 μg/m^3^ summer *vs.* 19.2 μg/m^3^ winter, *p* = 0.21) (Table 2).

**Figure 1:**
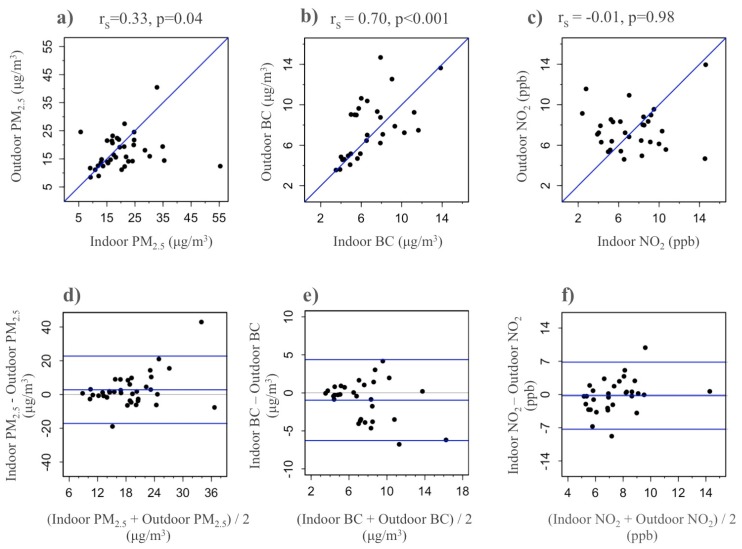
Agreement between indoor and outdoor residential levels of PM_2.5,_ black carbon, and nitrogen dioxide; Scatterplots of (**a**) PM_2.5_; (**b**) BC; and (**c**) NO_2_; Bland-Altman plots of (**d**) PM_2.5_; (**e**) BC; and (**f**) NO_2_.

Overall mean concentrations of indoor and outdoor pollutants (PM_2.5_, BC, and NO_2_) are statistically similar (Table 2). Furthermore, indoor and outdoor concentrations of both PM_2.5_ and BC were significantly correlated (PM_2.5_: r_s_ = 0.33, *p* < 0.04, BC: r_s_ = 0.70, *p* < 0.001), and strongly agreed according to the Bland-Altman plot (Figure 1). While indoor and outdoor concentrations of NO_2_ were not significantly correlated, the Bland Altman plot suggests high agreement and, importantly, an absence of systematic bias across pollutant means (Figure 1).

### 3.4. Allergens

Median concentrations of dog, cat, mouse, mite, and cockroach allergen from the bed were 194 ng/g, 133 ng/g, 162 ng/g, 5573 ng/g, 0.02 U/g, respectively. For dog, cat, and mite allergen, there were significantly higher levels in the bed compared to the floor (dog: 194 ng/g *vs.* 48 ng/g, *p* < 0.01; cat 133 ng/g *vs.* 1 ng/g, *p* = 0.05, and mite: 5573 ng/g *vs.* 543 ng/g, *p* < 0.001). There were no significant differences between bed and floor concentrations for the other measured allergens (Table 4).

All households had detectable mite allergen in bed samples, with 60% and 24% exceeding medium (2000 ng/g) and high (10,000 ng/g) threshold levels associated with increased risk of asthma sensitization and morbidity, respectively, according to threshold levels previously defined by Curtin-Brosnan [17] (See Table 4 and Section 2.6 for threshold definitions). Similarly, all floor samples had detectable levels of mite allergen, but only 8% met the medium threshold level and no floor concentrations met the high threshold level. Almost all homes had detectable levels of mouse allergen from the bed (96%) and floor (100%), with 24% and 16% of all bed and floor samples falling within the medium threshold range associated with allergic sensitization. Concentrations of mouse allergen met high threshold levels in 8% of floor samples (*n* = 2), but none of the bed samples.

Most homes had detectable levels of dog allergen (100% of bed and 76% floor), with 12% and 8% of bed and floor samples within the medium threshold range for sensitization, respectively. In contrast, only 80% and 48% of bed and floor samples had detectable cat allergens, and all were below threshold for sensitization. Finally, cockroach allergen was detected in the least amount of homes (48% of bed and 40% of floor samples), though 8% of bed samples and 8% of floor samples were at levels associated with increased risk of allergic sensitization. No dog, cat, or cockroach allergen samples exceeded high threshold levels.

**Table 4:** The characterization of indoor allergens from bed and floor dust samples using clinically relevant threshold levels.

Allergen	Threshold Categories ^a^			Total Detectable	Concentration	*p-*Value ^b^
	LOW	MEDIUM	HIGH		Median (IQR)	
**Dog (Can f 1)**					(ng/g)	
Bed	88%	12%	0%	100%	194 (107, 380)	<0.01
Floor	68%	8%	0%	76%	48 (27, 345)	
**Cat (Fel d 1)**					(ng/g)	
Bed	80%	0%	0%	80%	133 (43, 239)	0.05
Floor	48%	0%	0%	48%	1 (1, 169)	
**Mouse (Mus m 1)**					(ng/g)	
Bed	72%	24%	0%	96%	162 (122, 482)	0.37
Floor	76%	16%	8%	100%	125 (37, 437)	
**Mite (Der p 1)**					(ng/g)	
Bed	16%	60%	24%	100%	5573 (2676, 9539)	<0.001
Floor	92%	8%	0%	100%	543 (223, 1153)	
**Cockroach (Bla g 1)**					(U/g)	
Bed	40%	8%	0%	48%	0.02 (0.02, 1.2)	0.66
Floor	32%	8%	0%	40%	0.02 (0.02, 0.8)	

Notes: **^a^** Low threshold values based on allergen-specific limit of detection. Medium and high threshold levels were previously defined by Curtin-Brosnan *et al.* [14] and represent allergen concentrations thought to be associated with increased risk of allergic sensitization (medium) and increased asthma morbidity (high). All threshold categories are mutually exclusive: Can f 1, 78 ng/g (low), 2000 ng/g (medium), 10,000 ng/g (high); Fel d 1, 100 ng/g (low), 1000 ng/g (medium), 8000 ng/g (high); Mus m 1, 2 ng/g (low), 500 ng/g (medium), 1600 ng/g (high); Der p 1, 20 ng/g (low), 2000 ng/g (medium), 10,000 ng/g (high); Bla g 1, 1 U/g (low), 2 U/g (medium), 8 U/g (high); **^b^** Wilcoxon signed rank test of differences (bed *vs.* floor).

**Figure 2:**
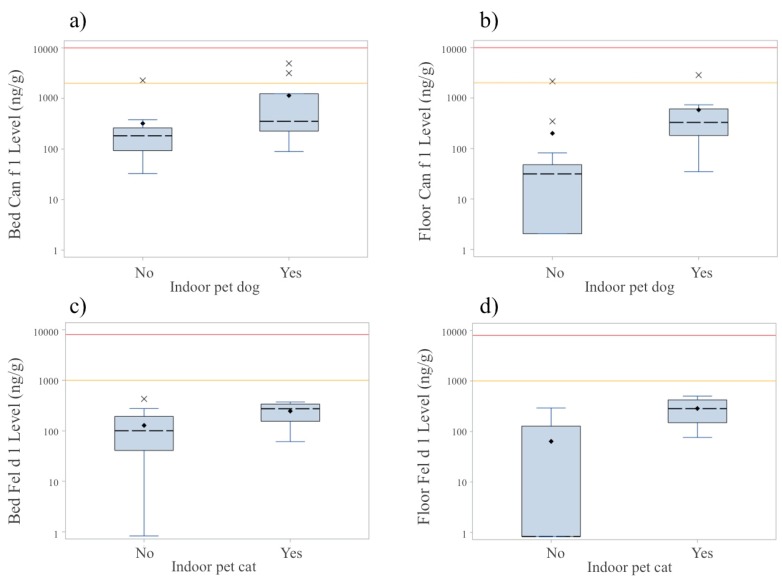
Concentrations of indoor (**a–b**) Can f 1 and (**c–d**) Fel d 1 allergens by the reported presence of indoor pet dogs and cats, respectively, from (**a**,**c**) bed dust and (**b**,**d**) bedroom floor dust samples. Boxes represent the interquartile range (IQR) of allergen concentrations and black dashed lines within the IQR correspond to median concentration values. Whiskers signify the lowest and highest values within 1.5 times the interquartile range, solid dots indicate mean concentrations, and “x” symbols are outlier values. Medium and high clinically relevant thresholds are represented by orange and red reference lines, respectively: Can f 1: 2000 ng/g (med), 10,000 ng/g (high); and Fel d 1: 1000 ng/g (med), 8000 ng/g (high).

As displayed in Figure 2, the distribution of indoor dog and cat allergen concentrations varies by the reported presence of indoor pet dogs and cats, respectively. Median bed and floor concentrations of Can f 1 in households reporting indoor pet dogs were significantly higher than concentrations in homes not reporting indoor dogs (bed: 351.8 ng/g *vs.* 182.3 ng/g, *p* = 0.026, and floor: 329.0 *vs.* 31.4 ng/g, *p* = 0.001). Median concentrations of Fel d 1 also tended to be higher in homes with indoor pet cats compared to homes without indoor cats (bed: 274.7 ng/g *vs.* 100.4 ng/g, *p* = 0.080; floor: 282 ng/g *vs.* 0.83 ng/g, *p* = 0.006). The reported outdoor presence of pet dogs or cats did not have a significant effect on measured indoor allergen concentrations (data not shown).

## 4. Discussion

We report that indoor household concentrations of PM_2.5_ and BC were similar to ambient concentrations in two peri-urban communities in Lima, Peru, suggesting that outdoor sources of pollution, such as traffic-related particles and gases, are significant drivers of domestic environmental exposures. Furthermore, while higher pollutant concentrations were observed near homes closer to main roadways compared to homes farther away, household characteristics did not significantly vary by road proximity. These results suggest that traffic-related air pollution is a significant contributor to indoor air quality in peri-urban Peruvian communities, and that homes near roadways are particularly vulnerable. Additionally, to our knowledge, our study is the first to characterize indoor levels of dog, cat, mouse, and cockroach allergens in Peru.

In contrast to tightly-sealed homes of industrialized regions where indoor activities, such as cooking, heating, and smoking, may have greater impacts on indoor pollutant levels than ambient sources [19], the Peruvian homes in this study exhibit a more open housing construction with high natural ventilation that may be responsible for greater contributions of traffic-related pollution to indoor environmental exposures. Our results highlight the significant influence of outdoor pollutant sources on indoor residential exposures in this environment, and indicate that future studies may be able to use outdoor residential measurements of PM_2.5_ and BC as surrogates for indoor pollutant concentrations. Interestingly, indoor and outdoor concentrations of NO_2_ were not significantly correlated, which is consistent with previous reports from urban areas in the United States [20]. It is possible that several known determinants of residential NO_2_—such as gas stove usage [20,21,22] as well as cooking time and ventilation patterns [21,22]—varied across households more so than sources of PM_2.5_ and BC, leading to inconsistent correlations between indoor and outdoor NO_2_ concentrations. Meanwhile, the Bland Altman plot suggested good agreement between overall mean concentrations of indoor and outdoor NO_2_ concentrations. Accordingly, research regarding respiratory diseases such as asthma in similar geographical regions should account for the strength and presence of both indoor and outdoor sources when assessing respiratory triggers within the home.

While published data on indoor pollutants in Peru is limited, prior reported averages of in-home concentrations of total PM in Lima were high, ranging from 9.0 to 159 μg/m^3^ (mean 43.4 g/m^3^) [10]. In the current study, we found weekly averages of indoor PM_2.5_—a component of total PM—to be 20 μg/m^3^, which is comparable to reports from mostly non-smoking, urban residences in North America [21,23,24]. Furthermore, concentrations of black carbon—a combustion-specific component of PM proposed to be a surrogate for diesel exposure in urban locations [25]—were similarly high in both indoor and outdoor residential areas (7.6 μg/m^3^ and 8.1 μg/m^3^, respectively). Together, these results support previous evidence that mobile source emissions, such as diesel traffic, are a dominant source of particulate pollution in urban areas, and in particular, may strongly influence indoor air quality in this peri-urban region of Peru.

Our finding that people living closer to the main roadways may be at greater risk of exposure to PM_2.5_, BC, and NO_2_ is consistent with the literature. Prior studies of traffic pollutants have often considered the effect of proximity to a single main roadway in urban locations [26,27], and distance to one or several roadways in well-gridded urban locations [25]. In contrast, the current study investigates the relationship between resident pollutant levels and road proximity in a peri-urban environment with a more complex roadway pattern. Our group previously reported a significant association between asthma symptoms and proximity to a single main roadway in this community, but not between total PM and roadway proximity [10]. The current study extends this research by further exploring the association of PM_2.5_ and other combustion-specific pollutants, *i.e.*, BC and NO_2_, with roadway proximity based on an expanded network of highly trafficked roads.. This is especially important with regards to BC, which has been previously identified as a more specific marker of traffic exposure compared to NO_2_ and PM_2.5_, based on BC’s more pronounced spatial gradients near roadways [25] and strong associations with respiratory health outcomes [28]. Our findings that residential BC levels are elevated in homes near main roads suggests that traffic-related BC is an important source of domestic pollutant exposure in this community.

We found seasonal variations in the degree of correlation between road proximity and air pollutants, particularly for BC and PM_2.5_. For example, during the summer season, which is characterized by high temperatures, low humidity, and rainfall, road proximity was significantly correlated with PM_2.5_, but not BC. This particulate gradient in the summer may be driven by non-exhaust traffic emissions, such as road dust generation and resuspension that are more common in drier seasons. In contrast during the winter, a significant correlation between road proximity and both BC and PM_2.5_ was identified. This may reflect the effect of winter rain and humidity on increasing deposition and decreasing residence time of combustion-related particulate pollution [29], resulting in steeper concentration gradients near the road. While these findings should be interpreted with caution due to low sample sizes, the presence of seasonal effects is likely [29] and warrants further investigation.

A novel aspect of this study was the characterization of indoor allergens within the home environment, which to our knowledge, has not been previously reported in peri-urban Peru. Mite allergen (Der p 1) was ubiquitous across households, mostly at levels previously demonstrated to be associated with increased allergic sensitization (>2000 ng/g) and morbidity (>10,000 ng/g) [17], and similar to studies from other humid coastal regions of Chile [30] and Brazil [31]. Similarly, mouse allergen was detected in almost all bedroom samples (96% of bed and 100% of floor samples), and at levels (>500 ng/g) previously associated with allergic sensitization [17] as well as increased morbidity and health care utilization [32] in approximately a quarter of participant homes. In contrast, cockroach allergen was detected in the least number of homes and at lower concentrations than previously reported in urban South America [31] and North America [24,33,34]; however, this may be due to sampling location, as detection of cockroach allergen in the bedroom may be several fold lower than the kitchen and living rooms [34]. Lastly, the limited cross-reactivity of Bla g 1 with other allergens potentially expressed by local cockroach populations, such as Per a 1 [35,36], may have led to an underestimation of total cockroach allergen exposure.

Meanwhile, we report relatively low percentages of indoor dog and cat allergens above relevant clinical thresholds (Table 4 and Figure 2); however, it is important to note that these results likely underestimate total exposure given the high prevalence of stray and tenant-owned dogs and cats that reside outdoors. Additionally, we found that households reporting the presence of pet dogs or cats indoors had substantially higher levels of Can f 1 and Fel d 1 allergens, respectively, compared to those without indoor pets. These results are in line with previous evidence from the literature [17] suggesting that participant reporting of pets may serve as a crude surrogate measure of increased exposure in the absence of direct measurements. Overall, the identification of indoor allergens at clinically relevant concentrations in relation to participant reports of exposure in this study could inform future studies aimed at determining their role in allergic and respiratory morbidity in these peri-urban regions.

Our pilot study has several limitations. First, the sample size of homes was small, limiting the ability to adjust for some potential confounders, such as individual housing characteristics. In addition, we did not specifically account for other exposure-related modifiers such as wind, traffic, and other social patterns of behavior (e.g., cooking, window-opening, smoking). While elements of these modifying factors are likely captured by season, random effects on indoor and outdoor pollutant concentrations within a season could further influence our results. In particular, accounting for cooking time, fuel usage, and ventilation patterns could help clarify the main determinants of indoor pollutants and explain the observed inconsistences in indoor-outdoor concentration patterns between NO_2_ and other traffic-related pollutants.

## 5. Conclusions

In summary, our results suggest that high concentrations of pollutants including PM_2.5_, BC, and NO_2_, are present within the homes of peri-urban communities near Lima, Peru, at levels similar to outdoor concentrations. This study highlights the importance of accounting for seasonal variability, multiple roadways, and combustion-specific particles, such as BC, in the characterization of spatial and seasonal trends of traffic-related air pollution. These exposures are higher in residences near main roadways, and suggest that traffic-generated pollution is a significant contributor to home exposures that are relevant to respiratory morbidity. In addition, indoor allergens, such as dust mite and mouse allergens in the bedroom, are present within these homes at levels that have the potential to worsen asthma morbidity. These findings are the first step in a series of environmental health assessments within a peri-urban region in Peru, which are essential in determining the factors that may influence the growing epidemic of chronic disease in this region.
